# Synchronized resistive-pulse analysis with flow visualization for single micro- and nanoscale objects driven by optical vortex in double orifice

**DOI:** 10.1038/s41598-021-87822-7

**Published:** 2021-04-29

**Authors:** Kichitaro Nakajima, Ryoji Nakatsuka, Tetsuro Tsuji, Kentaro Doi, Satoyuki Kawano

**Affiliations:** 1 Graduate School of Engineering Science, Osaka University, Toyonaka, Osaka 560-8531 Japan; 2 Graduate School of Informatics, Kyoto University, Sakyo-ku, Kyoto 606-8501 Japan

**Keywords:** Nanofluidics, Applied optics, Optical techniques, Fluid dynamics

## Abstract

Resistive-pulse analysis is a powerful tool for identifying micro- and nanoscale objects. For low-concentration specimens, the pulse responses are rare, and it is difficult to obtain a sufficient number of electrical waveforms to clearly characterize the targets and reduce noise. In this study, we conducted a periodic resistive-pulse analysis using an optical vortex and a double orifice, which repetitively senses a single micro- or nanoscale target particle with a diameter ranging from 700 nm to 2 $\mu$m. The periodic motion results in the accumulation of a sufficient number of waveforms within a short period. Acquired pulses show periodic ionic-current drops associated with the translocation events through each orifice. Furthermore, a transparent fluidic device allows us to synchronously average the waveforms by the microscopic observation of the translocation events and improve the signal-to-noise ratio. By this method, we succeed in distinguishing single particle diameters. Additionally, the results of measured signals and the simultaneous high-speed observations are used to quantitatively and systematically discuss the effect of the complex fluid flow in the orifices on the amplitude of the resistive pulse. The synchronized resistive-pulse analysis by the optical vortex with the flow visualization improves the pulse-acquisition rate for a single specific particle and accuracy of the analysis, refining the micro- and nanoscale object identification.

## Introduction

Identification of single particles with a micro- or nanoscale diameter, such as biological cells^1–4^, pollen allergen particles^5–7^, viruses^8–10^, and extracellular vesicles (i.e., exosomes)^11,12^, has attracted much attention for important applications, including environment surveys^13,14^ and medical diagnosis^15,16^. Resistive-pulse analysis using on-chip micro- and nanofluidic devices based on an ionic current^17–19^ or a tunnel current^20,21^ is a label-free method to analyze the characteristics of such particles; the target particles suspended in an electrolyte solution are introduced into a channel with a sensing structure such as a slit^22^, a pore^23,24^, or embedded counter electrodes with a small gap^25,26^, whose representative lengths should be comparable with the equivalent diameter of target particles. The translocation event of the particle across the narrowest part causes an abrupt current change that results in a resistive pulse. The resistive-pulse waveforms reflect the characteristics of specimens, such as volume^27^, aspect ratio^28^, and surface charge^29^. Indeed, previous works^30,31^ have demonstrated that the nanopore-based resistive-pulse analysis is a promising methodology for the detection and/or characterization of nanoscale materials. The waveform thus contributes to the identification of the specimens.

The amplitude of the resistive pulse becomes smaller as the particle diameter decreases, such as for nanoparticles and biological molecules, e.g., DNA. Then, the influence of noise significantly increases for these small particles. Thus, it is necessary to average the results from a sufficient number of waveforms to cancel out the noise, which is attributed to the Brownian motion of the target particles and intrinsic electrical noise, to achieve identification. However, the translocation event rarely happens, especially for low-concentration specimens, which increases the time required for acquiring enough signals to average them and obtain a reliable result. Furthermore, several forces, such as the electrophoretic force^32,33^ and the drag force from surrounding fluid flows caused by the pressure gradient and an electroosmotic flow^34–36^, act on the particle near the sensing structure, and they modulate the trajectory and velocity of the particle translocation. As a result, the particle motion in the sensing structure causes an apparent modulation of the ionic-current waveforms, deteriorating the reproducibility of the resistive pulse^37,38^.

In previous research^39^, several methods were proposed to control fluid flows using electroosmosis caused by the surface charges of micro- and nanochannel walls, which were expected to reduce the transport speed as target particles moved against the fluid flows. We also have conducted electrical measurements of nanoparticles using in-plane nanopores^7^ and nanopore channels^37,38^, by applying quadrupole electrodes and/or microscopic visualization with a transparent channel. Furthermore, slowing down the transport speed is helpful for a detailed analysis of a resistive pulse of targets that pass through a sensing structure, because the time resolution of the electric measurement is insufficient when the target molecules traverse a thin sensing portion at high speed; such circumstances are typical for the translocation of the DNA molecules across a nanopore membrane^19^. Herein, we propose a novel method to improve the reproducibility and signal-to-noise (S/N) ratio of the waveforms of specific single particles. This method will contribute to solving a common problem in the resistive-pulse analysis, in which the signal waveform buried in the noise component must be salvaged for nanoparticles, to identify single particles and molecules.

**Figure 1:**
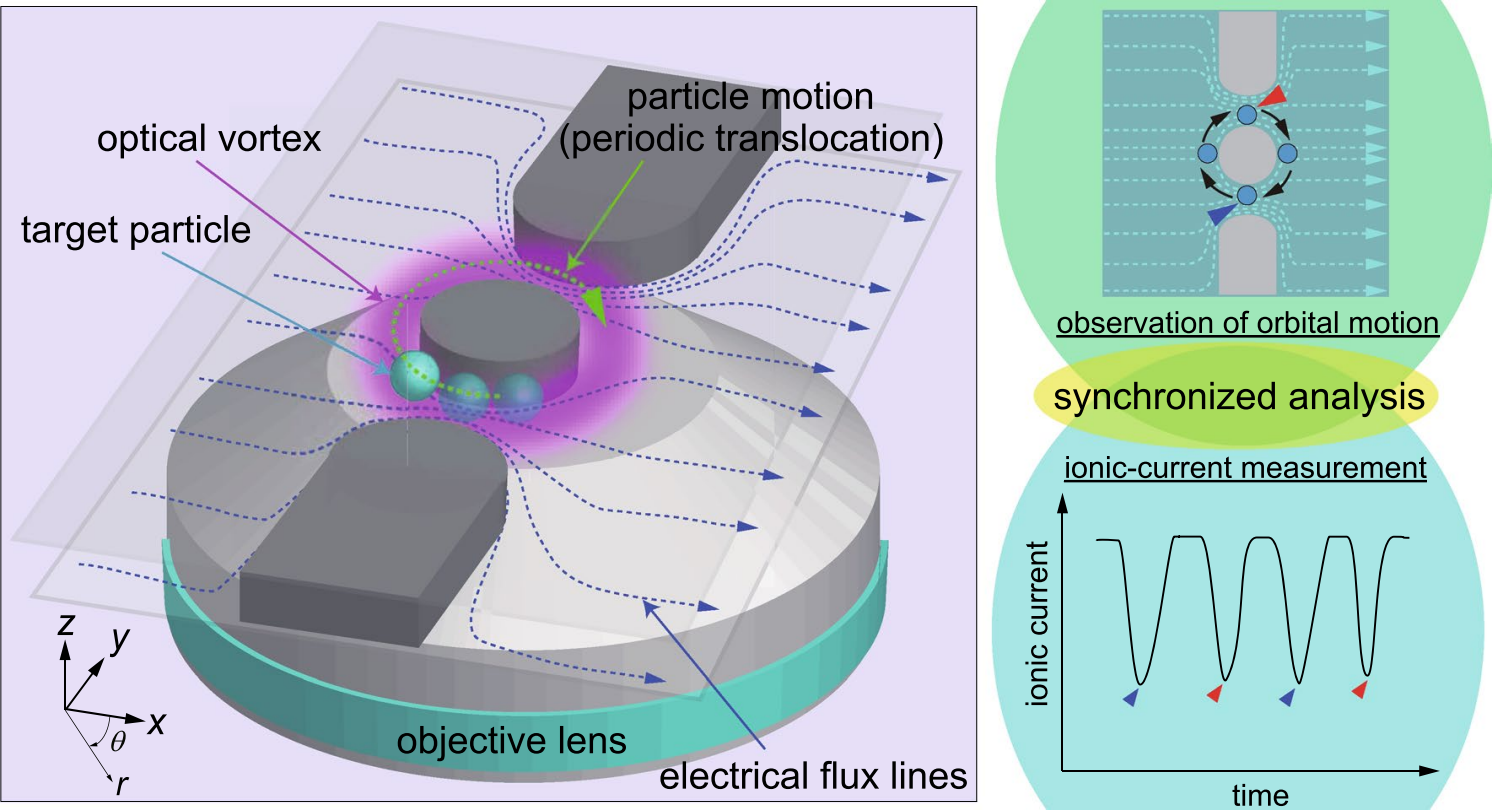
Concept of synchronized resistive-pulse analysis by an optical vortex in a transparent double orifice. Both the height and throat width of the orifice are 3 $\mu$m. (Details about the dimensions are described in the Supplementary Information.) An orbital motion of a single target particle is driven by the optical-vortex beam to periodically pass through the orifices. The translocation event is simultaneously observed by a microscope with an ionic-current measurement. The synchronized resistive-pulse analysis improves the S/N ratio of the resistive pulses by averaging a sufficient number of waveforms for a single particle. The coordinate system used in this study is shown in the left image.

In this study, we developed a synchronized resistive-pulse analysis method using an optical vortex with an in-plane double orifice for visualization of the target particles to improve the S/N ratio of the pulse waveforms. As shown in Fig. 1, a microchannel with an in-plane double orifice with a throat width of $\approx 3~\mu$m was fabricated as the sensing structure for the acquisition of resistive pulses of specific single particles. The transparent fluidic device with in-plane double orifice enables us to visualize a particle inside the channel to evaluate its trajectory associated with the electrical signals. Polystyrene (PS) particles with diameters ranging from 700 nm to 2 $\mu$m were selected as target particles to mimic dielectric biological particles, such as cells^1,2^ and pollen allergens^5^; the surface of the PS particles, which is widely investigated regarding its electrical properties, was negatively charged, as are the target biological particles.

Here, we adopted an optical vortex, discovered by Allen and his coworkers^40–42^, as a non-contact manipulator of the particles. The optical vortex possesses a helical wavefront along the propagation direction, a ring-shaped transverse intensity distribution at its focal plane^43^, and an orbital angular momentum around the optical axis. When a dielectric particle in a microfluidic channel is irradiated with an optical vortex, the particle is trapped in a circular orbit by an optical gradient force generated by the incident beam^44–46^. Furthermore, the particle is driven into the orbital motion and pushed against the upper wall of the channel because of an optical scattering force along the Poynting vector of the optical vortex^44–47^.

By optimizing the irradiation conditions for the optical vortex, single PS particles are manipulated to periodically traverse the double orifice embedded in a channel filled with an electrolyte solution, which leads to high-throughput acquisition of a sufficient number of resistive pulses for specific single particles. Previous works^48,49^ indicated that the repetitive acquisition of the pulses improves reproducibility and resolution of the resistive pulse analysis. Furthermore, we previously performed the repetitive pulse acquisition using a single-orifice device with a standard Gaussian optical tweezer^50^, which improved the S/N ratio of the waveform. The optical manipulation helps stable translocation of the target particles; near the micro- and nanoscale structure, the complex electrohydrodynamic flow is locally induced, often preventing the particle driven by the electrophoresis from entering the sensing portion. Even in such a situation, the optical tweezer can manipulate the target particles as they traverse the sensing portion.

In this study, we proposed another method to obtain high-resolution electrical signals of single particles focusing on the optical-vortex manipulation. Particle passing through the double orifice was constrained to a circular trajectory with a width on the order of tens of nanometers because the Brownian motion was suppressed by the optical gradient force in the radial direction, thus achieving a high repeatability of the resistive-pulse analysis. Furthermore, the pulses acquired from the translocation events were averaged to smooth the noise component and the pulse variation attributed to Brownian motion; the pulses were synchronously superposed with reference to micrographs of the particle motion.

We preliminarily analyzed the orbital motion of single PS particles driven by a focused Laguerre-Gaussian (LG) beam, which has been widely adopted as an optical-vortex beam in experimental studies^44–46^. The focused LG beam possesses a phase singularity on its optical axis and orbital angular momentum around the singularity which induces the orbital motion of particles irradiated with an LG beam^43^. We confirmed that a single PS particle, manipulated by the LG beam, periodically passes through the double orifice. Then, the ionic-current measurement and the microscopic observation were simultaneously performed to improve the S/N ratio. The amplitude of the acquired resistive pulse showed a correlation with the particle radius, which indicated not only that the pulses were mainly attributed to the volume exclusion of the particles, but also that the other factors affecting the pulse amplitude existed. The experimental results indicated that the change of the flow speed in the orifices modulates the resistive-pulse amplitude. We improved the S/N ratio by synchronizing the ionic-current measurement and the high-speed observation. In addition to the advantages in the resistive-pulse analysis, this study will provide an idea of applications of an optical vortex.

## Results and discussion

### Analysis of orbital motion of PS particle in a double orifice

**Figure 2:**
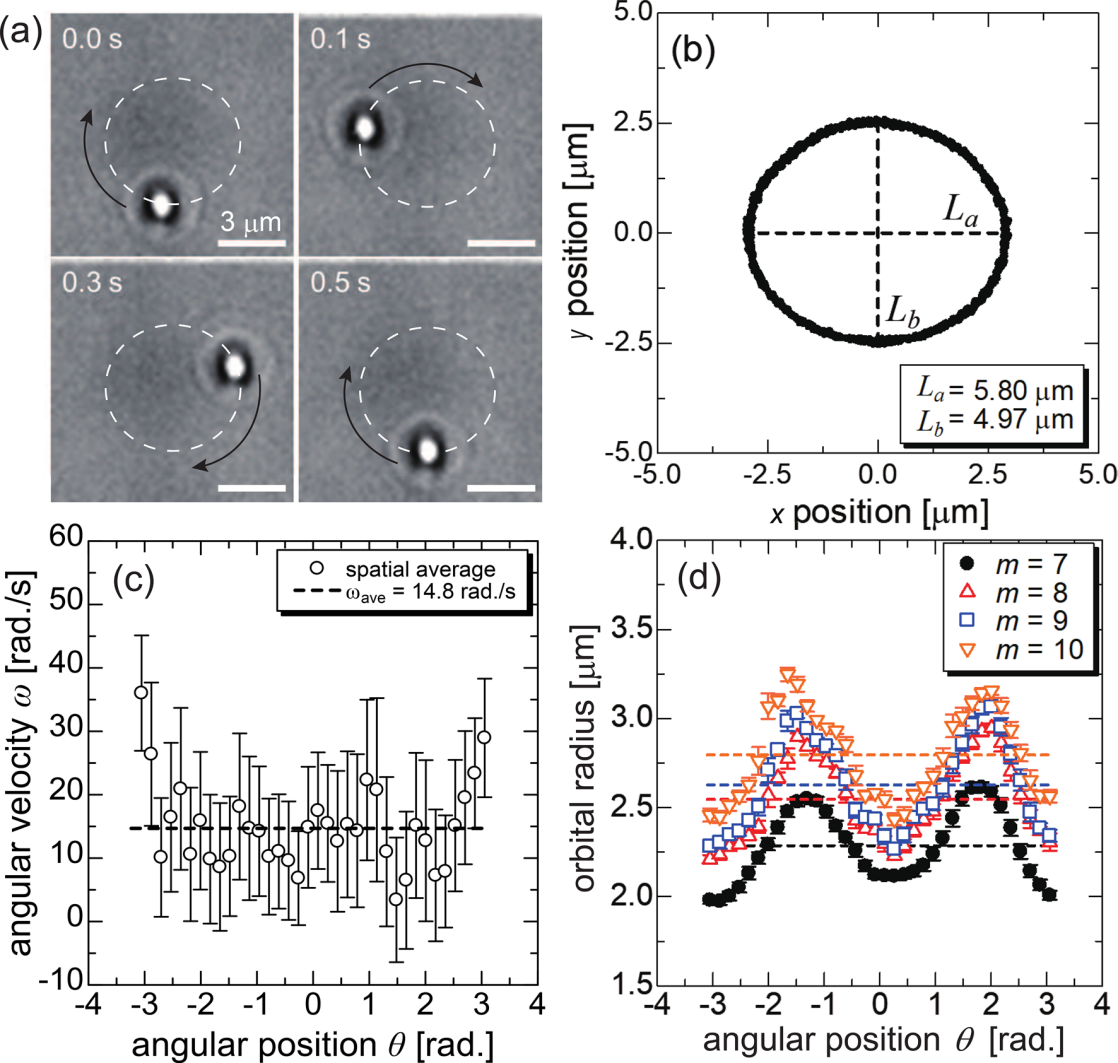
Orbital motion of single PS particle with a diameter of 1 $\mu$m driven by the LG beam with a topological charge $m=8$ in a channel without orifices. (**a**) Snapshots of the orbital motion. The white dashed ellipse represents the trajectory of the orbital motion. The scale bars denote 3 $\mu$m. (**b**) Trajectory of the orbital motion acquired by the particle tracking analysis. $L_{a}$ and $L_{b}$ denote the lengths of the major and minor axes, respectively. (**c**) Dependence of angular velocity on the angular position. Spatial average and error bars denote the average value and its standard deviation among each $0.1\pi$ rad, respectively. The $\omega _{ave}$ in (**c**) represents the average among all values. (**d**) Profiles of the ellipsoidal orbit driven by the LG beams with various values of *m* between 7 and 10. Each plot and corresponding error bar denote the spatial average and standard deviation of the particle trajectories.

We first analyzed the orbital motion of PS particles with a diameter, $d_{p}$, of 1 $\mu$m driven by the LG beam at an in-plane channel without an orifice as previously reported^47,51^. The appearance of the particle motion with a clockwise trajectory is shown in Fig. 2a. The intensity distribution for the LG beam depends on the topological charge *m*^52^, that is, as *m* increases, the vortex ring radius becomes larger^52^. We here apply an LG beam with $m=8$ to adjust the diameter of the orbital motion to a distance between the two orifices of $\approx 3$ $\mu$m, where the theoretical prediction of the orbital radius is consistent with the experimental results^46,47^.

In our experimental setup, the transverse intensity distribution in the *x*-*y* plane is slightly deformed from a perfect circle and results in the particle traveling with an ellipsoidal orbital motion, as shown in Fig. 2b; the deformation is attributed to the aberrations of the wavefront caused by an imperfection in the optical setup^53,54^. For example, Liang *et al.*^55^ reported that the orbital trajectory of 1-$\mu$m silica particles driven by an LG beam with $m=10$ had an elliptic shape, and its ellipticity, *e*, defined as $e=(L_{a}-L_{b})/L_{a}\times 100\%$ was 25.1%, where $L_{a}$ and $L_{b}$ denote the lengths of the major and minor axes of the ellipsoidal trajectory, respectively. In our setup, the ellipticity of the trajectory is 14.1% as shown in Fig. 2b, which is a reasonable value. The transverse intensity distribution also possesses a bias along the azimuthal direction^46,53,54^ defined in Fig. 1, leading to an angular position dependency of the angular velocity of the orbital motion indicated by the results of particle tracking analysis, as shown in Fig. 2c. As mentioned above, the orbital motion induced by the LG beam is not a perfectly uniform circular motion because of the experimental imperfection. However, the ellipsoidal trajectory and the angular-velocity inhomogeneity are not essential in the following ionic-current measurements because the trajectory and velocity of the orbital motion are invariant in a series of electrical sensing events except for the fluctuations caused by Brownian motion.

Because the fluidic device with a double orifice had the limitation of a fabrication accuracy of approximately 100 nm (see Supplementary Information), it was necessary to adjust the orbital radius of the particle manipulation on the order of hundreds of nanometers. We then evaluated the dependence of the orbital trajectory on *m*. As shown in Fig. 2d, the radial distribution of angular position shows a conformable profile for various values of *m* from 7 to 10. Meanwhile, the average radius of the ellipsoidal trajectory indicated by dashed lines in Fig. 2d increases in a positive correlation with *m* and changes in the range from 2.27 to 2.78 $\mu$m, which is enough to cover the range of the fabrication errors. For each *m*, the standard deviation of the trajectories at each point is approximately 30 nm, being $\approx$1% of the width of the orifice. The Brownian motion in the radial direction contributes to the noise of the resistive pulses^37,38^. The optical-vortex manipulation, which suppresses the undesired variation of the particle trajectory by the radial gradient force, will lead to determination of the accurate characteristics of target particles from the resistive pulses.

**Figure 3:**
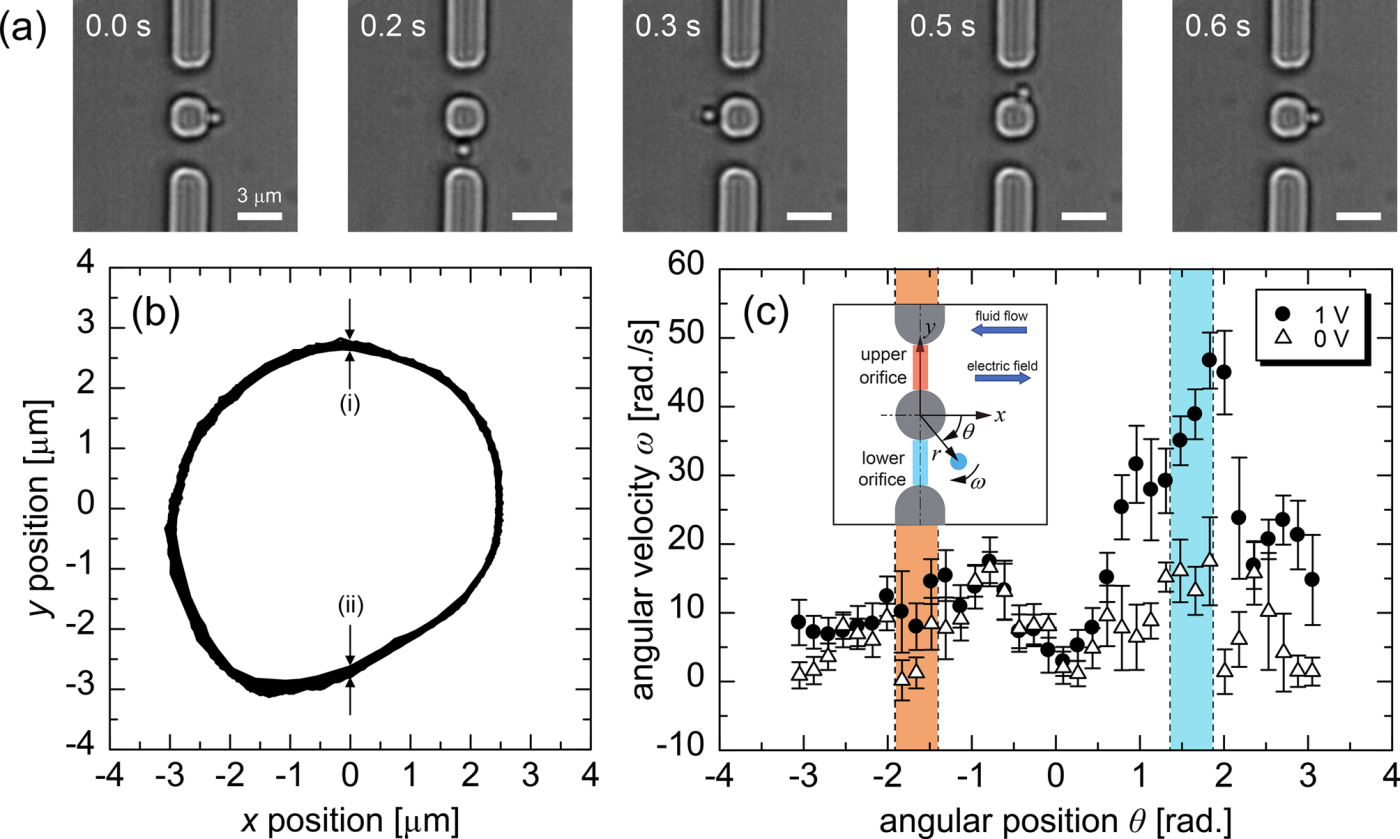
(**a**) Snapshots of the orbital motion of the 1~$\mu$ m PS particle in the double orifice under an applied voltage of 1 V. Scale bars denote 3 $\mu$m. A corresponding video is provided as Movie S1. (**b**) Trajectory of the orbital motion in the double orifice obtained by the particle tracking analysis. (i) and (ii) denote the width of the multiple trajectories in the upper and lower orifices, respectively. (**c**) Angular position dependency of angular velocity of the orbital motion with and without application of a voltage. Each plot and error bars again denote the spatial average and the standard deviation of the angular velocity. The areas colored red and blue correspond to the position of the upper and lower orifices, respectively. The inset shows the definition of each coordinate to the double orifice. In this case, the fluid flow and electric field point in a negative and positive *x* direction, respectively. The particle motion in the orifice is modulated by the external electric field and fluid flow with a speed of $O(10)~\mu$m/s, showing the different speeds between the upper and lower orifices.

Next, the orbital motion of a 1-$\mu$m PS particle was induced in the double orifices by an LG beam with $m=10$ as shown in Fig. 3a. To measure the ionic current, it is necessary to apply an external voltage across the double orifice in the *x* direction. We then analyzed the orbital motion under the application of a direct-current voltage of 1 V across the double orifice. Under the voltage application, a modulation of fluid flow was observed. Such a fluid flow has several components, such as pressure-driven and electroosmotic flows induced by applying the external voltage^34–36^, being the complex electrohydrodynamic phenomena. In addition, the PDMS channel is deformed by the laser irradiation to the microstructure through the local temperature increase^56^, which is often discussed as a drawback of the PDMS channel in the field of micro- and nanofluidic research. The deformation possibly disturbs the flow field inside the channel, especially near the double orifice, which will take a relatively long time to relax the deformation, i.e., to equilibrate the fluid flow. Because of the difficulties in controlling the electrohydrodynamic flow direction, it was randomly changed in the following experiments. Furthermore, the applied voltage induces an additional electrophoretic force on a particle^32,33^ in the orifice. As a result, the trajectory of the particle motion deformed as shown in Fig. 3b. The deformation is not observed in an in-plane channel without orifices in Fig. 2b, which is attributed to the fluid flow and external voltage.

The particle angular velocity in the double orifice was also analyzed as shown in Fig. 3c with and without application of the voltage. We then estimated the fluid flow speed in the orifices from the angular-velocity difference between the upper and lower orifices. It is important to estimate the flow speed because a flow of an ionic fluid with a net charge results in a streaming current. The flow speed $v_{f}$ was estimated as $v_{f} = r_{o}(\omega _{p}^{L} - \omega _{p}^{U})/2\approx 16.5$ $\mu$m/s, where $r_{o}, \omega _{p}^{L}$, and $\omega _{p}^{U}$ denote the average radius of the orbital motion, the angular velocities at the lower orifice, and the angular velocity at the upper orifice, in the case without application of a voltage. In addition, the particle average velocity was $\sim O(10^{-5})$ m/s, being of the same order as the magnitude of the fluid flow.

The electrophoretic effect results in further modulation on the particle velocity in the orifice on the order of 10 $\mu$m/s under the applied voltage of 1 V; the velocity of the electrophoresis, $v_{ep}$, is written as $v_{ep}=U_{ep}\times E$, where $U_{ep}$ and $E=O(10^{5})$ V/m denote the electrophoretic mobility of the particle and external electric field, respectively. The $U_{ep}$ is $U_{ep}=\zeta \epsilon _{0} \epsilon _{r}/\eta$ based on the Smoluchowski equation, where $\zeta =O(10^{-2})$ mV^50^, $\epsilon _{0}=O(10^{-12})$ F/m, $\epsilon _{r}=O(10^{1})$, and $\eta =O(10^{-3})$ Pa$\cdot$s denote the $\zeta$-potential of the particle, the permittivity of vacuum, relative permittivity and viscosity of surrounding liquid, respectively. As a result $v_{ep}$ is calculated as $v_{ep}=O(10^{-5})$ m/s, which is on the same order of that caused by the fluid drag force. The $O(10^{-5})$ m/s modulation of the particle velocity is also suggested by the different angular velocities between the voltage of 0 and 1 V in the lower orifice, as shown in Fig. 3c. The particle velocity modulation of the fluid flow on the order of $10~\mu$m/s are included in the subsequent experimental results from ionic-current measurements.

Moreover, the variation of the trajectories along the radial axis in the upper and lower orifices indicated by (i) and (ii) in Fig. 3b were 35.8 and 66.3 nm, respectively, remaining within a few tens of nanometers even under the fluid flow. Therefore, we consider that the gradient force of the optical vortex maintains an orbital trajectory even under fluid flow, which may highly influence the translocation velocity of the particle in the sensing structure. Although the orbital motion is affected by the fluid flow and external voltage, the LG beam manipulation succeeds in creating the periodic translocation of a single particle with a reproducible trajectory.

### Synchronized resistive-pulse analysis of micro- and nanoparticles

**Figure 4:**
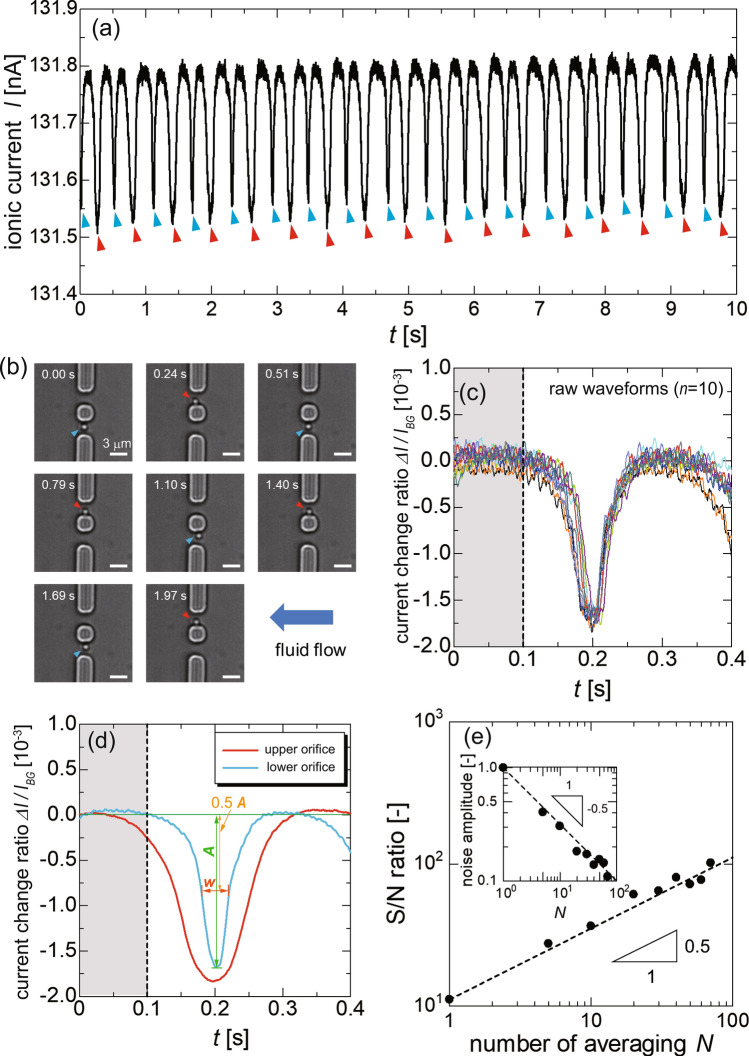
Results of the synchronized resistive-pulse analysis using the optical vortex for a single particle with a diameter of 1 $\mu$m under an applied voltage of 1 V. (**a**) Ionic-current, *I*, waveform acquired with the orbital motion through the double orifice. The red and blue arrows indicate the translocation of the particle through the upper and lower orifices, respectively. (**b**) Snapshots of the translocation of the particle through the double orifice. Scale bars in (b) denote 3 $\mu$m. (**c**) 10 typical waveforms corresponding to the translocation through the lower orifice, in which the ionic-current value is standardized by the background current. (**d**) Resistive-pulse waveforms averaged over 70 translocation events for the upper and lower orifices. *A* and *w* respectively denote the pulse amplitude and width which is defined as the full width at half maximum of the waveform. The gray area from 0 to 0.1 s indicates the waveform range used for the analysis of the S/N ratio. (**e**) S/N ratio dependency on the number of pulses *N* used in the synchronized averaging. The inset shows the decay of the noise amplitude as *N* increases. In keeping with the theoretical estimation, the noise amplitude of the waveform for *N* averaged pulses decays inversely proportional to $\sqrt{N}$, leading to improvement of the S/N ratio proportional to $\sqrt{N}$.

We performed the synchronized resistive-pulse analysis by visualizing a single particle with a diameter of 1 $\mu$m, as summarized in Fig. 4. The particle periodically passed through the double orifice with a frequency of approximately 1.7 Hz, as shown in Movie S1. Figure 4a shows an ionic-current waveform that was simultaneously acquired with the microscopic observation. This current drop is attributed to the exclusive volume of the particle within the orifice volume. The background ionic current, $I_{BG}$, was 131.8 nA at an applied voltage of 1 V. The change in the applied voltage affects the orbital motion of the particles in the double orifice through the electrophoretic effect, which often prevents a stable orbital motion. Thus, for each experiment, we selected the applied voltage value which allows us to acquire the current pulse with a stable orbital motion of the target particle. Also, we confirmed that the relative pulse amplitude with the different applied voltages remained unchanged, as shown in Fig. S3. We separately set the applied voltage in the following experiments. Meanwhile, it should be noted that the ionic current value when the trapped particle is out of the orifice is identical to that of the orifice without particles.

By the simultaneous observation of the orbital motion with the current measurement, as shown in Fig. 4b, we confirmed that the ionic-current drops indicated by the red and blue arrows in Fig. 4a correspond to translocation through the upper and lower orifices, respectively. As shown in Fig. 4a, the amplitude of the resistive pulse of each orifice was clearly different. The fraction of a resistive pulse $\Delta I$, which is an absolute value of the pulse amplitude, to the background current $I_{BG}$ was $\Delta I/I_{BG} = 1.70 \times 10^{-3}$ and $1.85 \times 10^{-3}$ for the upper and lower orifices, respectively. The width of the resistive pulse also differed between the translocation events through the upper and lower orifices. This difference in the pulse width is attributed to the difference in the angular velocity in each orifice, as illustrated in Fig. 3c. In this experiment, the fluid flowed in the negative *x* direction defined in the inset of Fig. 3c with a flow speed on the order of 10 $\mu$m/s, as discussed above. In contrast, the electric field induced by the applied voltage was in the positive *x* direction. In this case, the faster translocation speed of the particle resulted in a smaller amplitude of the resistive pulse. This point will be discussed later in this section.

The purpose of the synchronized resistive-pulse analysis with microscopic observation is to improve the S/N ratio of the pulse waveforms. The noise reduction leads to an accurate evaluation of the amplitude and width of the resistive pulses. For the synchronized analysis, the micrographs of the particle motion were analyzed to synchronously average the current pulses; we detected the translocation event as a brightness-value increase in each orifice through the image processing. A time $t=0$ in each electronic pulse was defined as the time when the brightness value reaches the maximum in each micrograph video. Then, the electronic pulses are superposed to decrease the noise component and analyze the resistive-pulse amplitude and time width, that is, the full width at half maximum. In this experiment, 70 translocation events were confirmed for each orifice by the high-speed observation. It took $\approx$35 s for the acquisition of 70 pulses. A typical set of 10 raw waveforms at the lower orifice and the waveforms averaged over 70 waveforms for each orifice are shown in Fig. 4c and 4d, respectively.

It should be noted that the resistive-pulse waveforms mainly include three kinds of waveform variation among the translocation events, specifically the variation of (i) the pulse width, (ii) the pulse amplitude, and (iii) intrinsic background noise of the electrical measurement system. Herein, the components of (i) and (ii) are essentially attributed to the Brownian motion of the target particle during the translocation event. The Brownian motion can be divided into the radial and azimuthal components, which are rigorously not independent components that influence one another. The latter component causes the variation of the pulse width indicated as *w* in Fig. 4d, leading to a variation with a coefficient of variation (CV) of 9.80% for the pulse width among the 70 pulses, that is, the average of $N=70$ pulses. Here, the CV is defined as the fraction of the standard deviation to the average value and is used as an indication of the reproducibility. In contrast, the Brownian motion along the radial direction caused the variation of the pulse amplitude indicated as *A* in Fig. 4d. The CV of the amplitude is 3.12% for $N=70$, which may be improved by the optical-vortex trapping force that restricts the radial Brownian motion. These fluctuations can be canceled out by averaging a sufficient number of waveforms. Here, it should be noted that the synchronized analysis of the pulse waveform with particle motion allows us to clarify the corresponding particle trajectory for each pulse. Although the trajectory dependency of the pulse was not detected in the microscale orifice, it may contribute to the investigation of the correlation between pulse amplitude and particle trajectory in the nanoscale experiment, where the effect of Brownian motion becomes larger.

The background noise component (iii) can also be suppressed by averaging. Here, we defined the background noise amplitude as $2\sigma$, where $\sigma$ is the standard deviation of the ionic-current value in the gray area, as shown in Fig. 4c, d, to analyze the S/N ratio of the pulse. In Fig. 4e, the S/N ratio for the pulse is plotted against *N* and shows an improvement as *N* increased. Based on the central limit theorem, a random-noise amplitude $2\sigma$ should decay in proportion to $N^{-\frac{1}{2}}$ to be $\approx 2\sigma /\sqrt{N}$ by averaging *N* pulses. In keeping with the theoretical prediction, the noise amplitude decays in the manner of $1/\sqrt{N}$ as shown in the inset of Fig. 4e. Nevertheless, the pulse amplitude remains unchanged within the synchronized averaging, which leads to an improvement of the pulse S/N ratio proportional to $N^{-\frac{1}{2}}$, as indicated by the dashed line in Fig. 4e. These experimental results represent the ability of the synchronized resistive-pulse analysis to reduce the noise component.

**Figure 5:**
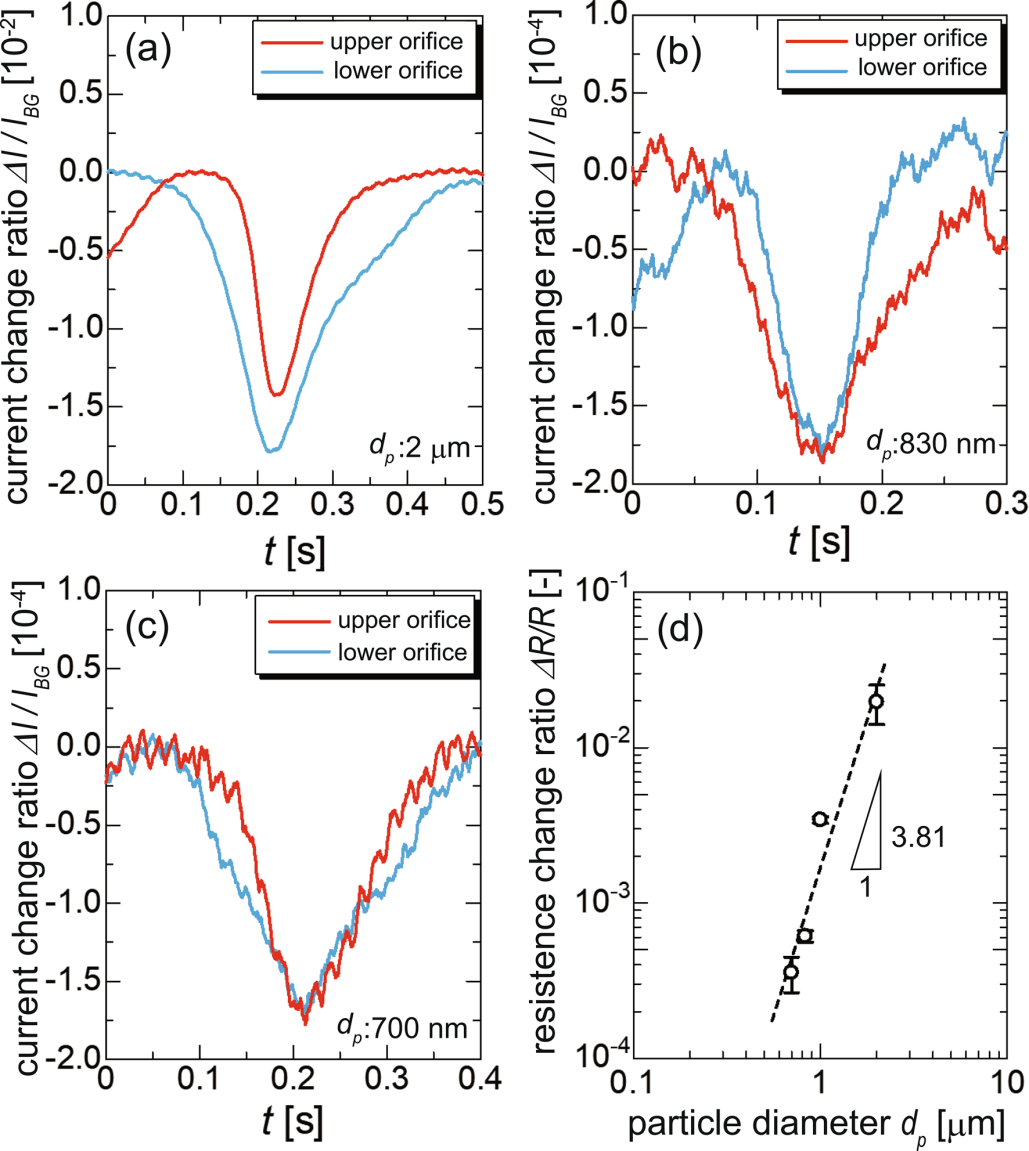
Results of resistive-pulse analysis using the optical vortex for particles with diameters of (**a**) 2 $\mu$m, (**b**) 830 nm, and (**c**) 700 nm. The applied voltages for the measurement of 2-$\mu$m, 830-nm, and 700-nm particles are 0.3, 0.5, and 0.6 V, respectively. The waveforms are the synchronized averages of 70 pulses. The vertical axis denotes the amplitude of the resistive pulse standardized by the background current. (**d**) Relationship between the particle volume and the amplitude of the resistive pulse, which is averaged over the upper and lower orifices. The slope of the fitted curve of 3.81 is not far from the prediction based on the exclusive volume of the particle (a slope of 3), equally indicating the existence of the other factors affecting the resistive-pulse amplitude. The error bars denote the standard deviation ($n\geqq 3$), where *n* is a number of the independently analyzed particle, not the number of averaging *N*.

To evaluate the accuracy of the periodic resistive-pulse analysis using the optical vortex, we measured the ionic current of particles with diameters of 700 nm, 830 nm, and 2 $\mu$m in addition to the 1-$\mu$m diameter discussed above. A goal of this experiment was to systematically investigate the relationship between the particle diameter and the amplitude of resistive pulses. The raw waveforms and snapshots of the orbital motion for each diameter are shown in the Supplementary Figures. Using the acquired waveforms corresponding to the particle motion, which was synchronously observed, we again shaped the synchronously averaged waveforms for both orifices, as shown in Fig. 5a–c, which correspond to diameters of 2 $\mu$m, 830 nm, and 700 nm, respectively. For all diameters, the presented waveforms are the result from averaging over $N=70$. For a diameter of 2 $\mu$m, the directions of the fluid flow and the electric field were in the negative and positive directions along the *x*-axis, respectively, as defined in Fig. 3c. The amplitude of the resistive pulse for the upper orifice where the particle moves against the flow is larger than that for the lower orifice, which shows the same tendency as that for 1-$\mu$m particles.

In Fig. 5b, c for the particles with diameters of 830 and 700 nm, although the waveforms are averaged, the noise component, whose amplitude is on the order of $10^{-5}$ to $10^{-6}$ in the background current, remains in the averaged waveform. The result of the fast Fourier transform of the averaged waveform, as shown in Fig. S7, revealed that the waveform of the 700-nm diameter particle includes a small noise component attributed to the electric power supply with a frequency of 60 Hz. However, any other systematic components were not observed for all particle diameters, which suggests that a noise on the waveform in the cases with 700- and 830-nm diameter particles essentially arises from the strong Brownian motion of the nanoparticles. Moreover, the amplitude difference between the upper and lower orifices is not observed in these cases. We will discuss this phenomenon later along with the cause of the amplitude difference.

When the double orifice is considered as its equivalent electric circuit (see Supplementary Note 1), the amplitude of the resistive pulse can be determined by the product of *C* and $\Delta R/R$, where *C* and $\Delta R/R$ denote the voltage-drop ratio in the double orifice and the resistance change ratio during particle translocation. The voltage drop ratio *C* is defined as the ratio of the voltage drop in the double orifice to the applied voltage. For double-orifice fluidic devices, voltage drops in the orifices depend not only on the dimensions of the double orifice, but also on the positions of the inlet and outlet holes where the Ag/AgCl electrodes are inserted. These holes were made by hand with a 3.5-mm hole punch, and thus precise positioning is difficult. The amplitude of the resistive pulses in Fig. 5a–c involves components of the other parts except for the volume exclusion at the double orifice and thus, the voltage-drop ratio is different for each particular fluidic device. To roughly compensate for the fabrication error among each device, we here used the equivalent electric circuit model of the double orifice, where we neglected the effects by a surface charge, an access resistance^57^, and so on. Although the measurement of various particles by a single device allows us to directly compare $\Delta I/I_{BG}$, we here used different devices to test whether a value of $\Delta I/I_{BG}$ has a reproducibility even using the devices which have the fabrication error.

We then evaluated the relationship between the particle volume and the resistance change ratio, $\Delta R/R$, associated with the particle translocation, as shown in Fig. 5d. The $\Delta R/R$ should be proportional to the particle volume due to a decrease in the electrical conductivity by the exclusive volume. The dashed line in Fig. 5d is a fitting line obtained by a power function, showing a slope of $\approx 3.81$. If the $\Delta R/R$ is only attributed to the exclusive volume of the particle, the slope of the fitted line should be $\approx$3. Also, it has been reported that a ratio of a particle diameter relative to an orifice dimension affects a resulting pulse amplitude^58^. If we assume the orifice as a cylinder with a diameter of $3~\mu$m, a slope of 3.28 is acquired by the theoretical prediction in the range of the particle diameter from 700 nm to $2~\mu$m. The slope of 3.28 is larger than that of a simple prediction with a volume exclusion but smaller than that of the experimental result. These facts indicate that parameters affecting the pulse amplitude except the exclusive volume are included in the pulse waveform. The local charge separation in the orifice, which is not considered in the theoretical model, should be one reason for the difference. Meanwhile, the difference possibly arises from the fact that the theoretical model assumes that the sensing portion has a single cylindrical geometry rather than a double orifice. However, the theoretical model suggests that the experimental gradient should be more than 3, which is qualitatively consistent with our experimental result. Consequently, we succeeded in distinguishing particles based on their diameter by reducing the noise component with a sufficient number of averaged synchronized pulse measurements.

Next, we focus on the difference in the resistive-pulse amplitudes between the upper and lower orifices. This difference may be problematic if a higher resolution for measuring a particle diameter is required, and thus the cause should be clarified. We systematically investigated the relationship between the resistive-pulse amplitude and the relative directions of the particle motion, electric field, and fluid flow, and resistive-pulse amplitude. In the optical-vortex manipulation, the rotation direction can be reversed by changing the sign of *m*, the orbital angular momentum, which enables us to change the direction of particle translocation through the orifices.

**Figure 6:**
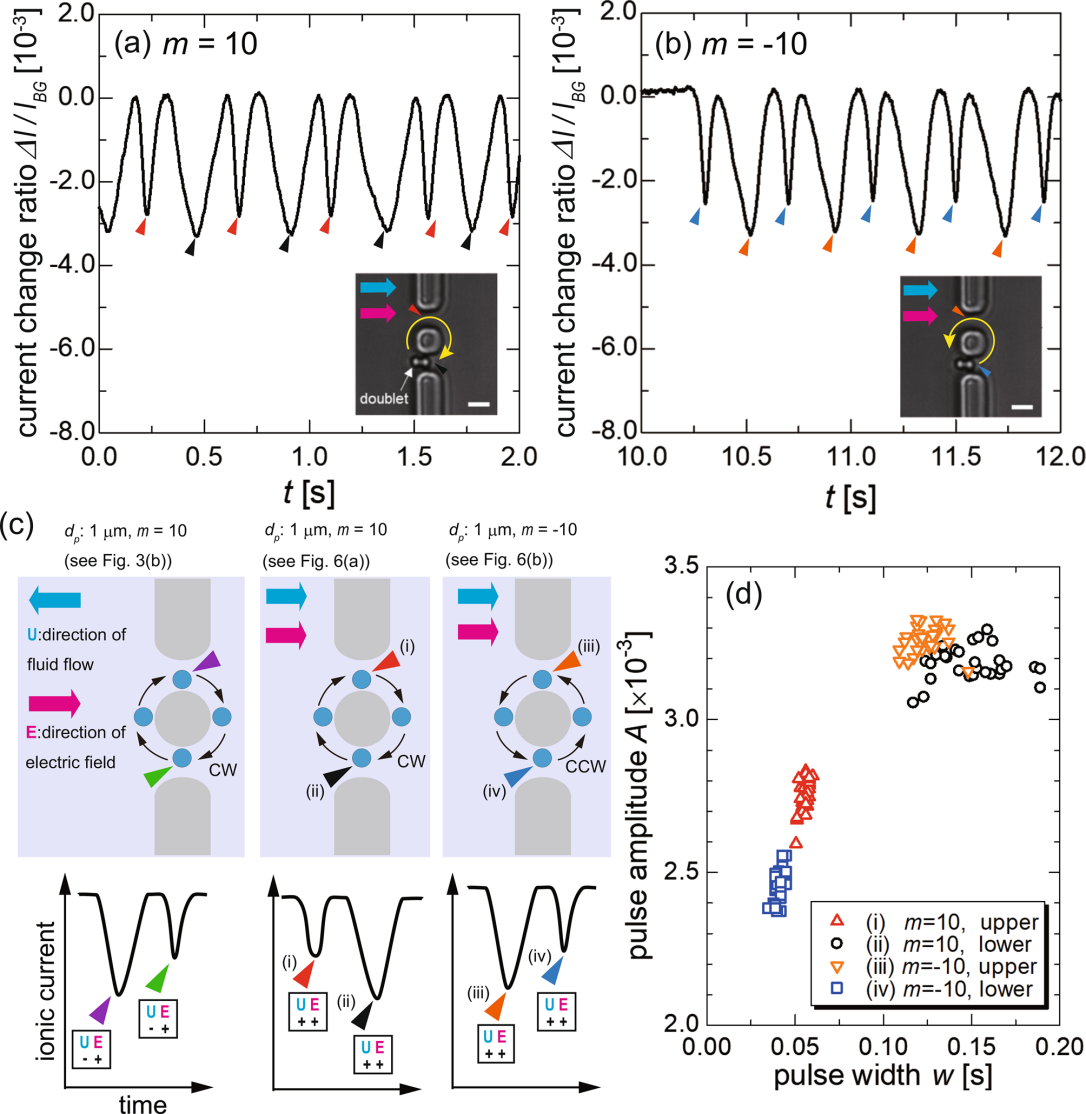
Resistive-pulse waveforms of the particle translocation driven by the LG beam with (**a**) $m=10$ and (**b**) $-10$. The ionic current is acquired with an applied voltage of 0.3 V. Insets show the appearance of the orbital motion in the double orifice. The blue, magenta, and yellow arrows represent the direction of the fluid flow, electric field, and orbital motion, respectively. The scale bars denote 3 $\mu$m. (**c**) Schematic summary of the relationship between the directions of the particle motion, electric field, and fluid flow, and the resistive-pulse waveforms. ${\mathbf {U}}$ and ${\mathbf {E}}$ denote the direction of fluid flow and electric field in the orifice, respectively. Here, the vector in a direction from left to light is defined as $(+)$. The abbreviations CW and CCW in the figure correspond to the rotation direction of the orbital motion being clockwise and counterclockwise, respectively. Here, it should be noted that the directions and velocities of the pressure-driven flow were uncontrolled, randomly changed for individual experiments, but were unchanged during the experiment. (**d**) Relationship between the pulse width and amplitude for a particle doublet. Note that the data for the 1-$\mu$m single particle are not plotted on this figure, because the absolute values of pulse width and amplitude were very far from the plotted region. Each plot color corresponds to the arrowheads in (**c**). The results indicate that a faster orifice translocation leads to a smaller pulse amplitude based on a change in the relative velocity between the particle motion and fluid flow.

As shown in the insets of Fig. 6a,b, a doublet of 1-$\mu$m particles, which is a chain of two particles, was adopted as the target to clearly capture the difference in the pulse amplitude between the upper and lower orifices: in the experiment, single particles escaped from the optical-vortex trapping immediately after the sign of *m* was reversed due to the small trapping stiffness. The experimental result is shown in Movie S2, in which the surrounding fluid flows in the positive *x* direction defined in Fig. 3c. The resistive-pulse waveforms resulting from $m = 10$ and $m=-10$ in a single run of the experiment are presented in Fig. 6a and 6b, respectively. The directions of the particle motion and the fluid flow are schematically depicted in the insets of Fig. 6a and 6b, respectively. The amplitude of the resistive pulse again shows a difference between the upper and lower orifices. For $m = 10$, the doublet periodically traversed the orifices in a clockwise manner, and then the doublet moved against the flow direction in the lower orifice. As a result, the amplitude of resistive pulses obtained at the lower orifice was larger than that at the upper orifice. Conversely, for $m = -10$, the resistive pulse amplitude became larger at the upper orifice than at the lower orifice. These results are consistent with the tendency seen in Fig. 4.

We summarize the dependency of the resistive pulse amplitudes on the directions of the particle motion in the orifice, the electric field, and the fluid flow, in Fig. 6c. The results indicate that the amplitude of the resistive pulses is independent of the relative direction of the particle motion to the electric field, ${\mathbf {E}}$. However, the amplitude of the resistive pulse of a particle moving against the fluid flow is always larger than that of a particle following the fluid flow. Moreover, it is confirmed that the difference between the amplitudes does not mainly originate from the difference in the orifice dimensions due to the fabrication error. Then, we analyzed the dependency of the amplitude on the width of the resistive pulses, that is, the time to transit the orifice, in case of the particle doublet. Figure 6d shows the relationship between the amplitude and the width for 30 pulses for each case labeled as (i) to (iv). This scatter plot indicates that the resistive pulse of particles that move against the fluid flow, (ii) and (iii), shows amplitudes clearly larger than those of particles that follow the flow, (i) and (iv). Furthermore, the resistive pulse of case (i) shows slightly larger amplitudes and larger widths compared with those of case (iv). These results confirm that the orifice transit time of the particle has a positive correlation with the pulse amplitude by the mechanism discussed in the next paragraph.

It is inferred that the difference in amplitude is attributed to the modulation of the advection of ions; that is, in the micro-orifice, the electrolyte solution is positively charged due to the presence of an electric double layer. The fluid flow, including the pressure-driven and electroosmotic flows, actually brings a net positive charge across the orifices, affecting the resulting ionic-current value. Here, the pressure-driven and electroosmotic flows are considered together because of difficulty in separating each other, where the only electroosmotic flow actually transports the net charge. The change of ionic-current density caused by the modulation of the advection speed is evaluated as $\Delta I_{ad}~\sim ~\Delta u c'$, where $\Delta u$ is the modulation of the flow-advection speed due to the particle translocation and $c'$ is the net charge density of the ionic solution present in the orifice. Because the ionic solution adopted in this study is electrically neutral, the net positive charge in the orifice may be mainly attributed to the electric double layer formed by the countercations attracted to the orifice surface and PS particle surface. We assume that the orifice wall and PS particle surface, which have negative $\zeta$-potentials, induce the positive charges^59^ in the orifice with a density of $c'~=~O(10^{3})$ C/m$^{3}$ (the estimation process is described in Supplementary Note 2). Then, it follows that the fluid flow in the orifices modulated by the particle motion, $\Delta u$, is on the order of 100 nm/s. Here, the $\Delta u$ in the orifice is invisible in our experimental system. Thus, we assumed that the modulation effect was a few percent relative to the original fluid flow with speed on the order of 10 $\mu$m/s. As a result, $\Delta I_{ad}$ is estimated between $O(10^{-3})$ and $O(10^{-4})$ A/m$^{2}$.

Such a small ionic-current density, corresponding to *O*(10) fA in this experiment, is quite difficult to measure accurately. However, the visualization enables us to quantitatively evaluate the particle velocity and translate it to a current difference. The background ionic current should be mainly associated with electrophoresis of the ions, $I_{ep}$, which is estimated to be on the order of *O*(10) A/m$^{2}$ using experimental parameters. (Details are also in Supplementary Note2.) Thus, the amplitude modulation of the resistive pulse caused by the advection effect, $\Delta I_{ad}$, is smaller than $I_{ep}$, by a factor of $10^{-4}$. This estimation is consistent with the difference in amplitude observed in the experiments as shown in Fig. 4d, where the difference between the pulse amplitudes of the upper and lower orifices is $(1.70-1.85)\times 10^{-3}=O(10^{-4})$. The rough estimation of the advection effect on the pulse-amplitude difference is that a faster velocity of the particle motion relative to the fluid flow results in a larger modulation of the fluid flow in the orifice, which transports the net charge caused by the electric double layer, leading to a change in the pulse amplitude. Furthermore, this theoretical estimation, resulting from the coordination of the ionic current measurement and the particle tracking analysis, supports the contribution of the advection effect to the amplitude difference, although it is much smaller than that of electrophoresis. It should be noted that, by considering the advection effect as described above, we could determine its importance in the resistive-pulse analysis by controlling the particle motion with the optical vortex. Thus, the accuracy of the fluid flow control technique may be an essential experimental parameter toward a higher detection precision, and it remains as our future work.

## Concluding remarks

We developed a synchronized resistive-pulse analysis using an optical vortex in a double orifice to improve the yield and accuracy of the resistive-pulse analysis for single micro- and nanoscale objects, which are affected by strong Brownian motion. Target particles were manipulated by an optical vortex to periodically traverse the double orifice and to suppress their Brownian motion. This manipulation achieved the high-throughput acquisition of resistive pulses for a specific single particle with high repeatability. By this method, the S/N ratio of the waveforms was improved in proportion to the square of the number of averaged pulses, which was enabled by the simultaneous observation of the orbital motion in the transparent fluidic device. For each waveform, the reproducibility of the width and amplitude was 9.80% and 3.12% in the case of the particle diameter of $1~\mu$m, respectively, according to their CVs. These sufficiently small variations are attributed to accurate control of the particle velocity and trajectory by the optical forces. The discriminability of the synchronized resistive-pulse analysis was demonstrated for a variety of particle diameters. It succeeded in distinguishing particles with diameters of 700 nm, 830 nm, 1 $\mu$m, and 2 $\mu$m by the amplitude of the resistive pulse. In addition, our experimental results provide a novel insight into the contribution of fluid flows to the resistive-pulse amplitude.

In this study, we fabricated a transparent double orifice with an orifice gap of 3 $\mu$m and used an LG beam with an azimuthal mode in the range from $m = 7$ to 10. The device provided a variety of orbital radii in the orbital motion of target particles by changing the topological charge of the optical vortex and the optical setup. These adjustments can be suitably made depending on the size of the target particles. For the application of this method into biological particle identification, we need to downsize the device further to the nanoscale, where the effect of the particle surface charge density should be one of the dominant factors on the resistive pulse. Thus, our future work will apply the periodic resistive-pulse analysis by the double-orifice fluidic device to a nanoscale experiment to distinguish biological nanoparticles, such as pollen allergens and viruses.

## Methods

### Fabrication of the double-orifice fluidic device

The transparent double-orifice fluidic device was composed of a flat quartz-glass slide ($24 \times 36$ mm$^2$, No.1, Matsunami Glass Co. Ltd., Osaka, Japan) and a polydimethylsiloxane (PDMS) block that has a channel pattern on its surface. The PDMS was purchased from Dow Corning Toray Co., Ltd.(Sylgard$\textregistered$184, Japan). The PDMS block is made with a silicon mold fabricated by photolithography and a reactive ion etching process as previously described^46^. Dimensions of the PDMS block are shown in Fig. S8a.

Holes with a diameter of 3.5 mm were made in the PDMS block with a biopsy puncher (BP-25F, Kai Industries Co., Ltd., Japan) for setting electrodes to measure the ionic current. The glass slide was washed by ultrasonication for 10 min. The PDMS block and the glass slide were irradiated with an excimer laser (SVK111R-1N1-NF0, USHIO, Japan) for 2 min, modifying the surfaces to be hydrophilic. The PDMS block and the glass slide were then bonded by heating them at 200 $^\circ$C for $\sim 2$ min. The appearance of the device is shown in Fig. S8b.

### Optical setup

In this study, we constructed an optical system that irradiated PS particles with an optical vortex for simultaneous observation. Figure S8c is a schematic illustration of the optical setup. A continuous-wave semiconductor laser with a wavelength of 1, 064 nm (ASF1JE01, Fitel, Furukawa Electronics, Japan) was adopted as the light source. The continuous-wave Gaussian beam from the light source passed through the half-wave plate to align the direction of polarization. Then, the Gaussian beam was transformed into an LG beam, which is the optical vortex used in this study, by a liquid-crystal-on-silicon spatial-light modulator (X13138-03, Hamamatsu Photonics K. K., Japan) with computed holograms. The generated LG beam entered an optical system consisting of an inverted microscope (IX-71, Olympus, Japan) with an oil-immersion objective lens with a magnification of 40 ($\mathrm {NA}=1.40$, UPLXAPO40XO, Olympus, Japan). The laser power after the objective lens was measured by a power meter (3A-QUAD, Ophir Optronics, Jerusalem, Israel), and it was approximately 300 mW. This laser power was enough for the particle manipulation; higher laser power sometimes generated bubbles inside the channel and thus was avoided. The device was placed on an autostage and irradiated with the focused LG beam for manipulating the PS particles.

For observation of the particle motion in the fluidic device, the device was irradiated with transmission light from a halogen lamp (U-HGLGPS, Olympus, Japan). The transmission light was observed by the objective lens, which is identical to that for the LG-beam irradiation, and a complementary-metal-oxide semiconductor camera (Zyla 5.5, Andor Technology, Northern Ireland). The frame rate of the microscopic observations was usually set to be 100 fps. Especially for the measurement of the displacement of the Brownian motion, the frame rate was set to be 1, 000 fps. The direction of the pressure-driven flow was judged by a wide field observation which observed a field of view as shown in Fig. S1a. By the observation, we confirmed that the direction of the fluid flow was unchanged during the experiment.

### Ionic-current measurement

We built the ionic-current measurement system as shown in Fig. S8c. We inserted a pair of handmade spiral Ag/AgCl electrodes into the holes of the double-orifice fluidic device as shown in Fig. S7b. A direct-current voltage was applied to the electrodes by electric supply equipment (PMX18-5A, KIKUSUI, Japan). We used an ammeter with a measurement accuracy of $\sim 1$ pA (VersaSTAT 4, AMETEK, USA). The sampling rate was set to 2 kHz. To decrease the noise component, the fluidic device and the electrodes were covered by a laboratory-built electric shield gauge.

### Preparation of the particle solution

Information about the particles is summarized in the Supplementary Information. The stock solution was diluted by a solution containing 1-mM KCl and 1 vol% Triton-X 100. The concentration was adjusted to be $4\times 10^{-3}$ vol%. Before introducing the solution into the fluidic device, the solution was irradiated with ultrasonic waves for $\sim 10$ min to disperse the aggregated particles, if any, into single particles.

## Supplementary information

Supplementary material 1 (mp4 856 KB)

Supplementary material 2 (mp4 1686 KB)

Supplementary material 3 (pdf 972 KB)
