# Supplementary material for: Synchronized resistive-pulse analysis with flow visualization for single micro- and nanoscale objects driven by optical vortex in double orifice
Source: Sci Rep. 2021 Apr 29;11:9323. doi: 10.1038/s41598-021-87822-7 (PMC8085213; doi:10.1038/s41598-021-87822-7)
Supplement: Supplementary file 3 — Supplementary material 3 (pdf 972 KB) [file 41598_2021_87822_MOESM3_ESM.pdf]

Supplementary Information

Synchronized resistive-pulse analysis with flow visualization for single micro- and nanoscale objects driven by optical vortex in double orifice

Kichitaro Nakajima, Ryoji Nakatsuka, Tetsuro Tsuji, Kentaro Doi, and Satoyuki Kawano

Supplementary Note 1: equivalent electric circuit of the double orifice

We here describe the equivalent electric circuit of the double-orifice fluidic device to discuss the amplitude of the resistive pulses in the experiments. First, we show the entire fluidic device in **Fig. S1(a)**. The double orifice is located at the center of the image. The structure is schematically illustrated in **Fig. S1(b)**. On both sides of the orifices, there are channels with square pillars to prevent the channel roof from collapsing. The hole for introduction of the particle suspension is positioned further away from the double orifice. Ag/AgCl electrodes were inserted into these holes to apply voltage to the channel. The resulting assumed equivalent electric circuit is schematically illustrated in **Fig. S1(c)**, where R and $R_c = R_c^1 + R_C^2$ denote the equivalent electric resistance of a single orifice and a channel with pillars, respectively. Based on this circuit, the voltage-drop ratio, C, in the double orifices is described as follows:

$$C = \frac{R}{R + 2R_c}. \qquad (1)$$

R is determined by the dimensions of the orifice structure as

$$R = 2\rho \int_0^{\frac{w}{2}} \frac{dx}{S(x)}, \qquad (2)$$

where ρ, w and $S(x)$ denote ionic-current conductivity of the solution, the orifice length, and the sectional area of the orifice in the *y*-*z* plane at each *x* position, respectively. By substituting the dimensions into **Eq. (2)**, R is calculated as $R = 2.83 \times 10^{-1}\ \mathrm{m}^{-1} \times \rho\ \ \Omega \cdot \mathrm{m}$. However, R_c depends on the positions of the holes, specifically the number of pillar row, n, and is written as $R_c = 6.39 \times 10^{-2}\ \mathrm{m}^{-1} \times n\rho\ \Omega \cdot \mathrm{m}$. Note that R_c was calculated using the pillar dimensions, as shown in Fig. S1(d). Because n is not the same for all of the fluidic devices that we used, we summarize n as C, which is estimated by **Eq. (1)**, and the amplitude of the resistive pulses as $\Delta I/I_{BG}$ in the experiments for particles with diameters of 700nm, 830 nm, 1 μm, and 2 μm in **Table S1**.

Based on the circuit in **Fig. S1(c)**, the amplitude of the resistive pulses is described as follows:

$$\frac{\Delta I}{I_{BG}} = C\left(\frac{R}{R'} - 1\right), \qquad (3)$$

where R' denotes the electric resistance of an orifice containing a particle. From **Eq. (3)**, the resistance change ratio, $\Delta R/R$, is described as follows:

$$\frac{\Delta R}{R} = \frac{R' - R}{R} = -\frac{\frac{1}{C}\frac{\Delta I}{I_{BG}}}{1 + \frac{1}{C}\frac{\Delta I}{I_{BG}}}. \qquad (4)$$

Eq. (4) can be used to compensate for the amplitude of the resistive pulses for each diameter for each corresponding resistance change ratio of the vertical axis in **Fig. 5(d)**.

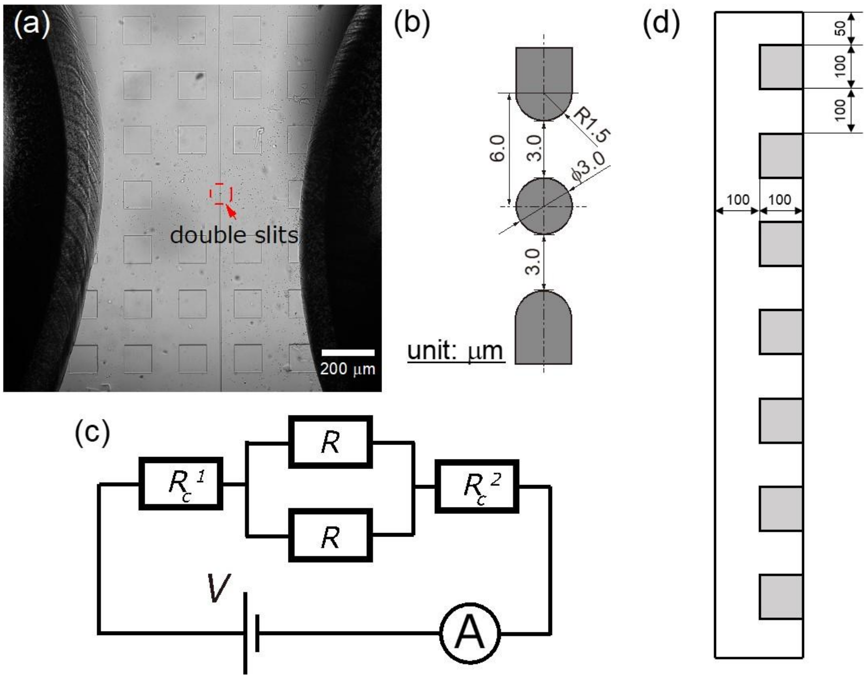

Fig. S1. (a) Micrograph of a double-orifice fluidic device. The number of rows of pillars, n, is determined from the micrograph. (b) Dimensions of the double-orifice that were used for the theoretical estimation. (c) Schematic illustration of the equivalent electric circuit of the double-orifice fluidic device. (d) The dimensions used for the calculation of R_c.

Table S1. Values for the number of pillar row n, voltage drop in the double orifice C, and the amplitude of the resistive pulse $\Delta I/I_{BG}$ in the experiments with four particle diameters.

Diameter (μm)	n	C (%)	$\Delta I/I_{BG}$ (-)
0.70	6	42.47	-1.51×10^{-4}
0.83	11	28.70	-1.75×10^{-4}
1.00	4	52.54	-1.80×10^{-3}
2.00	3	59.95	-1.18×10^{-2}

Supplementary Note2: estimation of the pulse amplitude modulation

Here, we describe the order estimation of the amplitude modulation based on the advection effect, ΔI_{ad} , and the background ionic-current density attributed to the electrophoresis of ions, I_{ep}. First, the modulation of the pulse amplitude was estimated from the change in the flow speed of the electrolyte solution in the orifice. Usually, the ionic-current density caused by the advection of an electrically non-neutral liquid, I_{ad}, is written as $I_{ad} = nzeu$, where n, z, e, and u denote the number density of the ions, the valence of the ion species, the elementary charge, and the speed of fluid flow, respectively. Here, we estimate the change in the ionic-current amplitude caused by the advection-speed change, Δu, which is attributed to the relative speed between the particle motion and the fluid flow. This current-density change, ΔI_{ad}, is written as $\Delta I_{ad} = \Delta u c'$, where Δu and c' denote the change of the fluid speed in the orifice and the net charge included in the orifice which is composed of the anions, cations, and electric double layer formed on the surfaces of the channel and the particle. We assumed that the Δu is on the order of 100 nm/s, which is a few percent against a background flow speed of $O(10)$ μm/s. Furthermore, based on Ref. 51, we hypothesized that the solid surfaces, that is, the PDMS channel walls and PS-particle surfaces, possess a surface charge with a density of $\sigma = O(10^{-2})$ C/m^2. Based on these assumptions, ΔI_{ad} [A m^{-2}] is estimated as follows

$$\Delta I_{ad} = \frac{\sigma S}{V} \cdot \Delta u \sim O(10^{-3}) \text{ [A m}^{-2}], \qquad (5)$$

where, n_{K^+} and n_{Cl^-} are the number density of potassium ions and chloride ions, respectively; S is the surface area of the orifice and particle; and V is the volume of the electrolyte solution inside the orifice, where they are calculated on the assumption that the particle is 1 μm in diameter and the orifice is cubic with a side length of 3 μm.

In contrast, the ionic-current density caused by the electrophoresis of the ions, I_{ep}, is written as

$$I_{ep} = \frac{Dze}{k_B T} cE \sim O(10^{1}) \text{ [A m}^{-2}], \qquad (6)$$

where $D \sim O(10^{-9})$ m^2/s, $z = 1$, $e = 1.60 \times 10^{-19}$ C, $k_B = 1.38 \times 10^{-23}$ J/K, $T \sim 3.00 \times 10^{2}$ K, $c = 1.00 \times 10^{-3}$ M = 6.02×10^{23} m^{-3}, and $E = O(10^{4})$ V/m respectively denote the diffusion constant of the ions, the valence of the ions, the elementary charge, Boltzmann constant, absolute temperature, the concentration of the ions, and the applied electric field across the orifice. The values in Eqs. (5) and (6) are used in the discussion in the main text.

Supplementary Information1

Table S2. Dimensions of the double orifice for 10 devices. The notations *a*, *b*, and *c* correspond to each dimension in **Fig. S2**. The dimensions were measured by a laser microscopy system (OLS4100, Olympus, Japan). Note that the microfluidic devices used in the measurement of the dimensions were not used in the resistive-pulse analysis experiment because the surface of the channel was possibly contaminated by the microscopic measurement procedure.

Device number	a (μm)	b (μm)	c (μm)
1	3.01	3.00	2.63
2	3.26	2.88	2.63
3	2.50	3.26	3.38
4	2.88	2.63	2.50
5	2.38	3.63	3.63
6	3.38	2.50	2.50
7	2.38	3.38	3.26
8	2.50	3.38	3.51
9	3.13	2.75	2.88
10	3.00	2.50	2.50
**average**	**2.84**	**2.99**	**2.94**
**standard deviation**	**0.35**	**0.38**	**0.43**

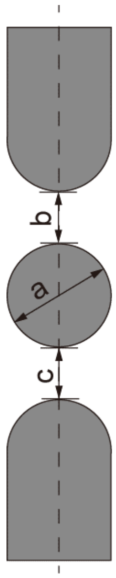

Fig. S2. Dimensions of the double orifice.

Supplementary Information2

Table S3. Information on the PS suspensions.

particle diameter [μm]	manufacturer	product number
0.70	Thermo Fisher Scientific	G700
0.83	Merck	F080
1.00	Molecular Probes	F8823
2.00	Molecular Probes	F8827

Supplementary Figures

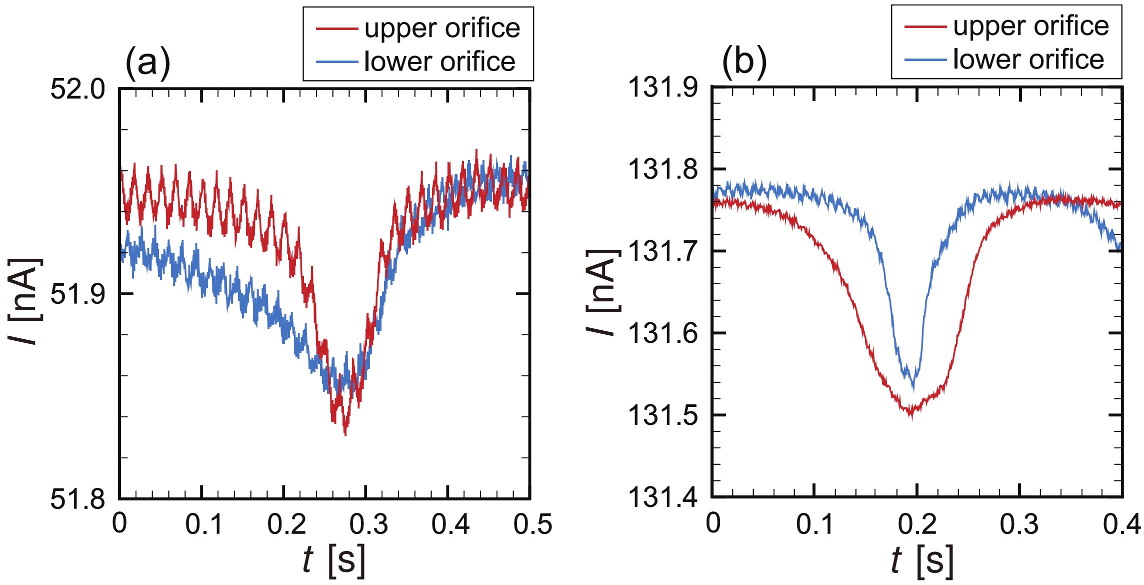

Fig. S3. The resistive pulse of the particle with a diameter of 1 μm under the applied voltages of (a) 0.5 and (b) 1 V. The relative pulse amplitudes to the background current are 2.02×10^{-3} ((a), red), 1.73×10^{-3} ((a), blue), 1.97×10^{-3} ((b), red), and 1.75×10^{-3} ((b), blue), respectively. Although the pulse deforms along the horizontal (time) axis due to the change in the electrophoretic force on the particle, the pulse amplitude remains unchanged in both upper and lower orifices.

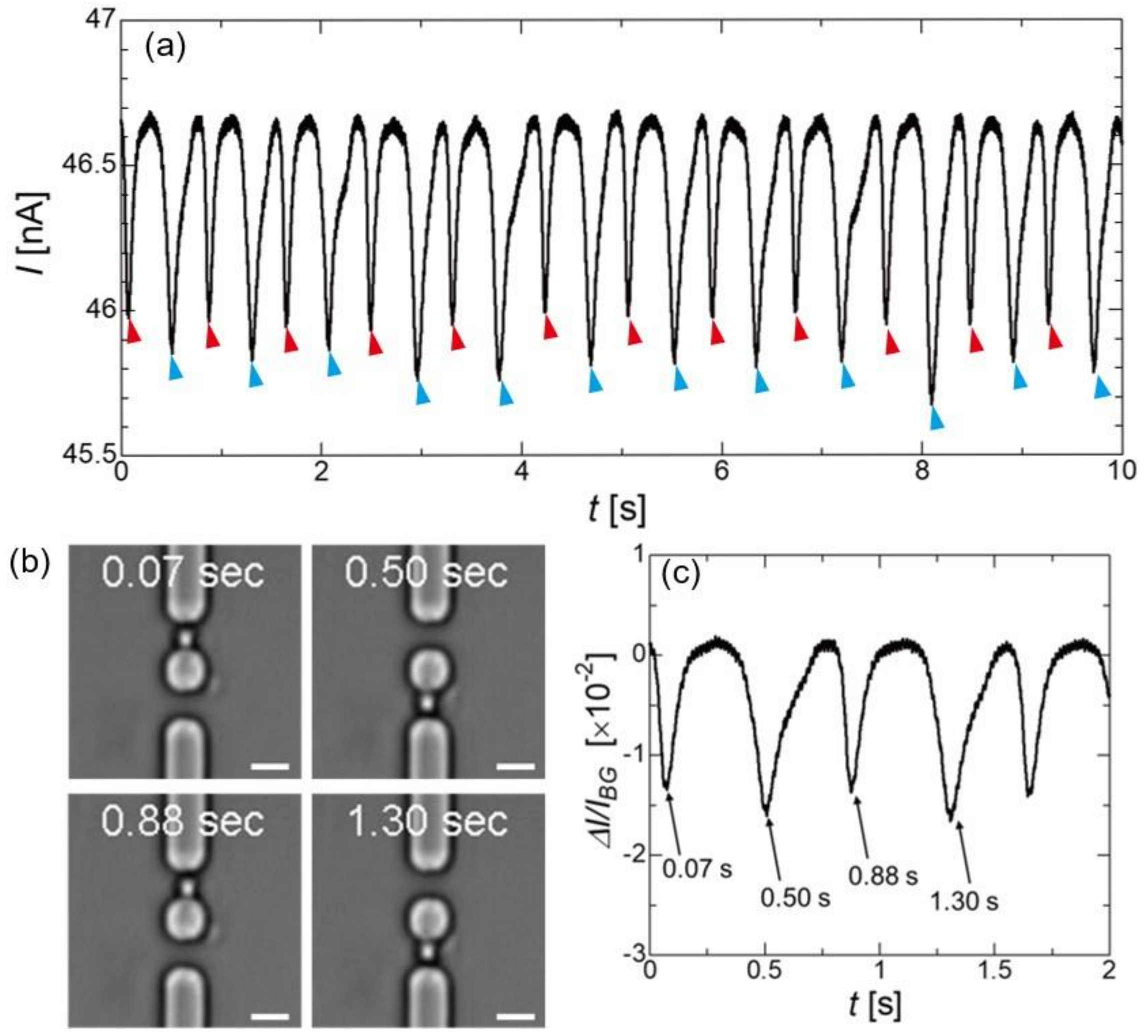

Fig. S4. Results of the synchronized resistive-pulse analysis of a single particle with a diameter of 2 μm. (a) Resistive-pulse waveform acquired from orbital motion in the double orifice. The red and blue arrows indicate the translocation of the particle through the upper and lower orifices, respectively. (b) Snapshots of the translocation event of the particle. (c) The resistive-pulse waveform corresponding to the snapshots in (b).

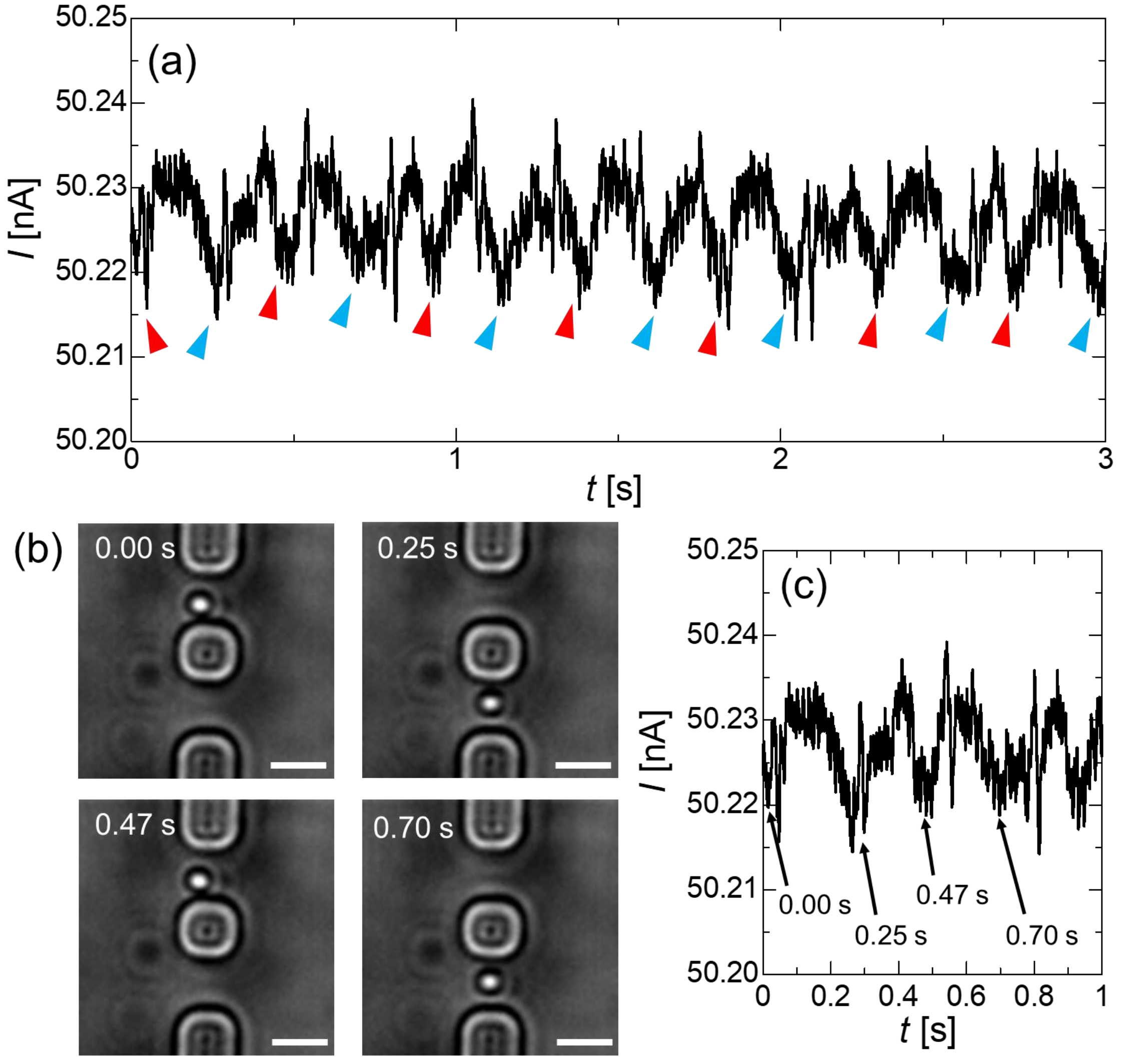

Fig. S5. Results of the synchronized resistive-pulse analysis for a single particle with a diameter of 830 nm. (a) Resistive-pulse waveform acquired from the orbital motion through the double orifice. The red and blue arrows indicate the translocation of the particle through the upper and lower orifices, respectively. (b) Snapshots of the particle translocation event. (c) The resistive-pulse waveform corresponding to the snapshots in (b).

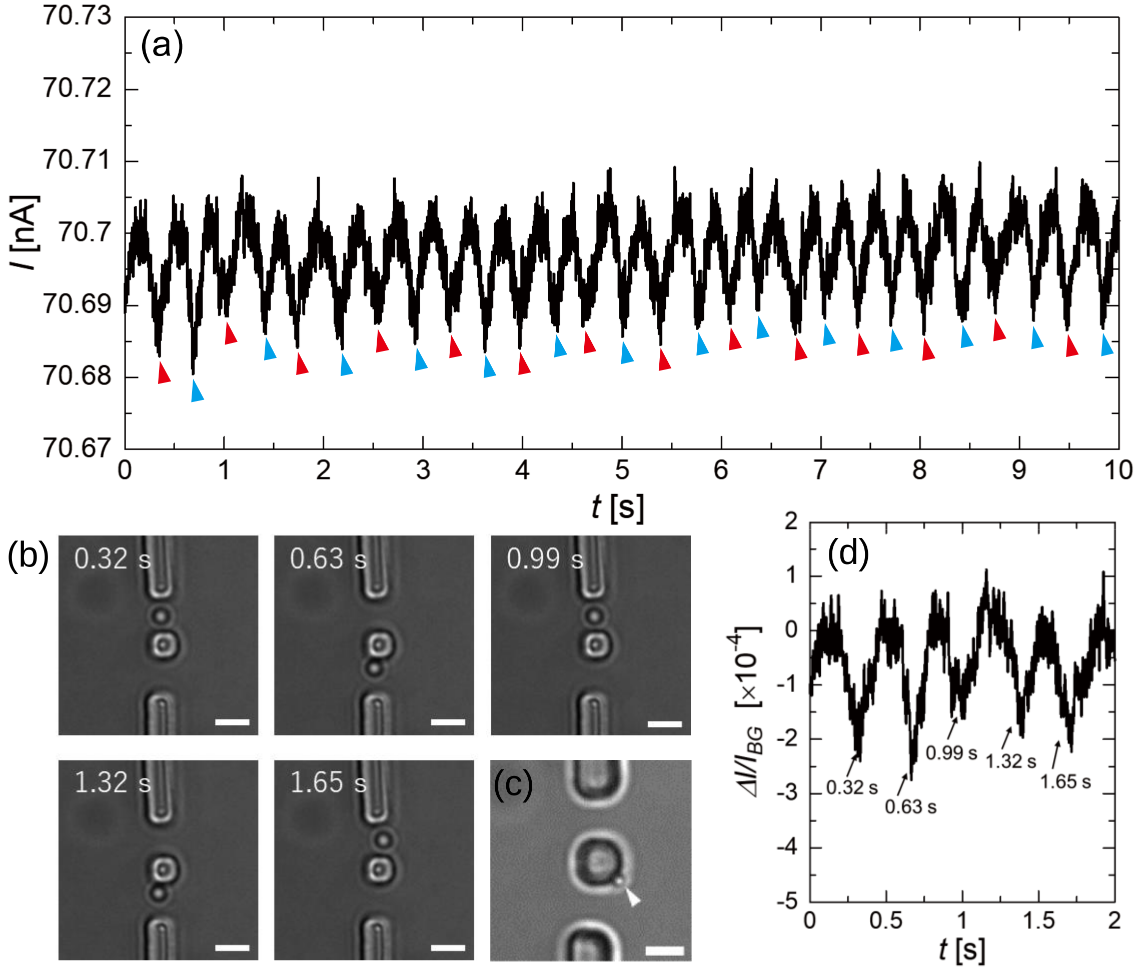

Fig. S6. Results of the synchronized resistive-pulse analysis of a single particle with a diameter of 700 nm. (a) Resistive-pulse waveform associated with the orbital motion of a single particle. The red and blue arrows indicate the translocation of the particle through the upper and lower orifices, respectively. (b) Snapshot of the orbital motion of the particle. Note that the snapshots were obtained with the objective lens out of focus to drive the stable orbital motion through the double orifice. The particle, thus, seems larger than its actual size. (c) Snapshot of same particle as in (b), obtained at the focal plane of the objective lens (i.e., in focus). Scale bars in (b) and (c) represent 3 μm. (d) Resistive-pulse waveform corresponding to the snapshots in (b).

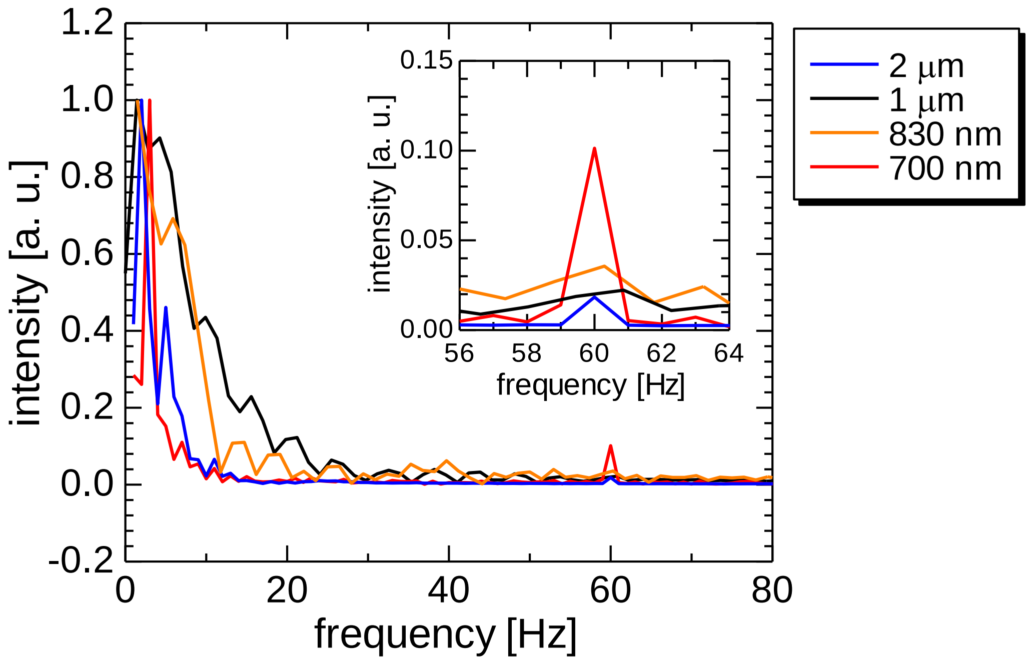

Fig. S7. The fast Fourier transform spectra of the acquired resistive-pulse waveforms for different particle diameters after the synchronized average (*N* = 70). Inset is an enlarged view near 60 Hz, showing that the waveform of a 700-nm diameter particle includes a small 60-Hz noise component attributed to an electric power supply.

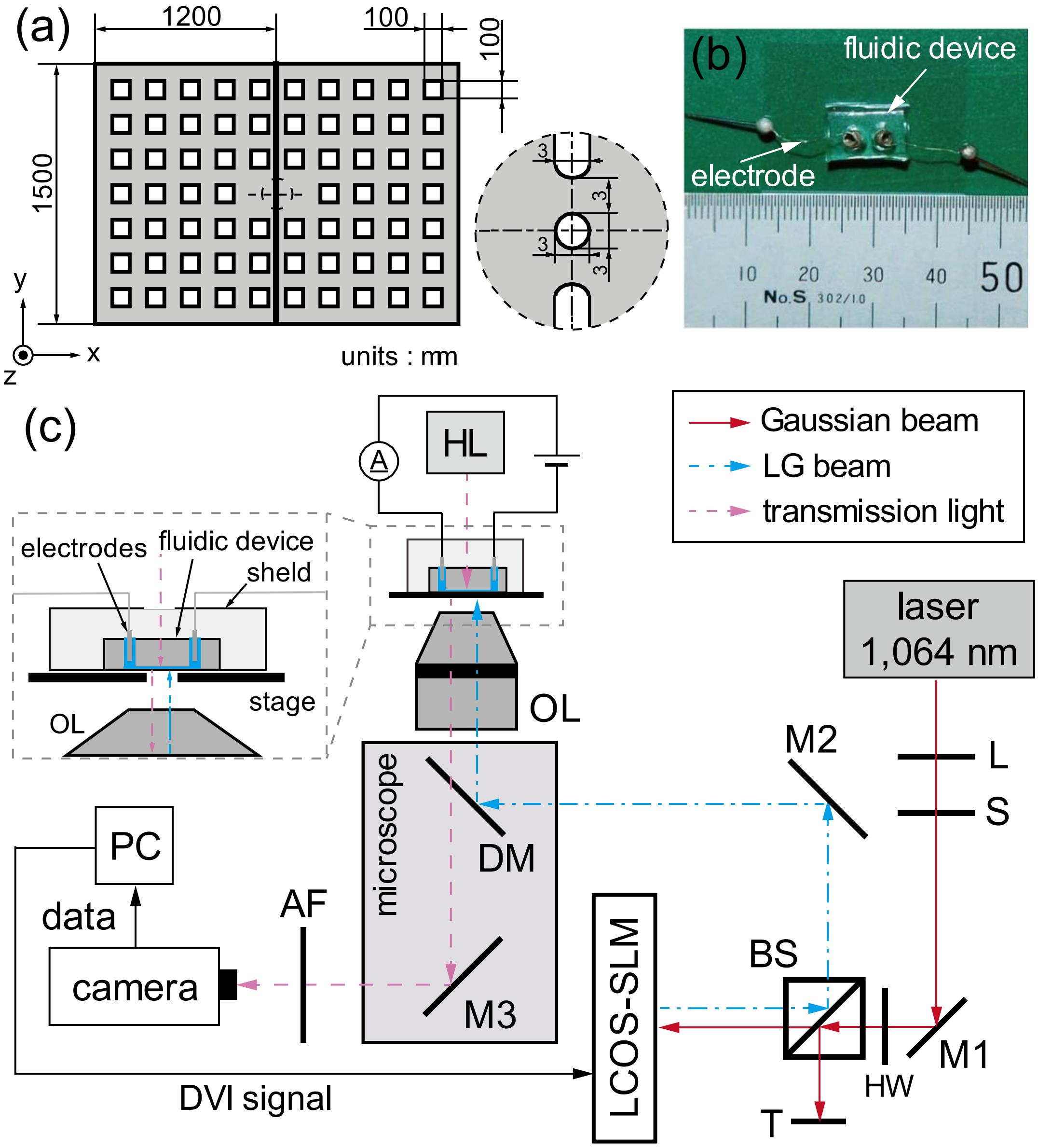

Fig. S8. (a) Dimensions of the double-orifice fluidic device in a PDMS block. The double orifice is placed at the center of the device. An enlarged view of the double orifice is provided in the dashed circle. (b) Appearance of the double-orifice fluidic device after a pair of the electrodes were inserted. (c) Schematic illustration of experimental setup for the optical manipulation by the LG beam in the double-orifice fluidic device. Abbreviations in (c) are as follows: L, lens to collimate the beam; S, mechanical shutter; M, mirror; BS, beam splitter; T, terminal; LCOS-SLM, liquid-crystal-on-silicon space-light-modulator; HW, half waveplate; DM, dichroic mirror; OL, objective lens; and HL, halogen lamp.

Movie Captions

Movie S1. Orbital motion of single particle with a diameter of 1 μm through the double orifice.

Movie S2. Rotation reversal experiment in the double orifice for a doublet composing particles with a diameter of 1 μm. The topological charge was reversed from $m =$ 10 to $m =$ -10 at 6.6 s.
